# Diagnostic Value of Emission Computed Tomography Combined with Computed Tomography for Metastatic Malignant Tumor of Spine

**DOI:** 10.1155/2022/5847589

**Published:** 2022-05-26

**Authors:** Feng Qin, Yapei Feng, Panpan Zhang, Yuemei Li, Weiqiang Fan

**Affiliations:** ^1^Department of Spinal Surgery, Dongying Shengli Oilfield Central Hospital, Dongying City 257100, Shandong Province, China; ^2^Department of Reproductive Medicine, Dongying Shengli Oilfield Central Hospital, Dongying City 257100, Shandong Province, China

## Abstract

**Objective:**

To explore the diagnostic value of emission computed tomography (ECT) combined with computed tomography (CT) for metastatic malignant tumor of spine.

**Methods:**

By means of retrospective study, a total of 102 patients with extraskeletal primary malignant tumor treated in our hospital from February 2019 to February 2021 were selected as the subjects. All patients had single lesion of the spine, of which 72 were malignant and 30 were benign according to the results of pathological examination. ECT and CT examinations were performed to all patients, and by taking the pathological findings as the gold standard, the sensitivity, specificity, positive predictive value and negative predictive value of ECT, CT, and their combination were calculated, and their efficacy in diagnosing metastatic malignant tumor of spine was analyzed.

**Results:**

A total of 68 (94.4%) metastatic malignant spinal tumors were detected by ECT combined with CT, with a detection rate of 100% in breast cancer and lung cancer, 94.1% in liver cancer, and 78.6% in prostate cancer, respectively; the combined diagnosis had a diagnostic sensitivity of 94.4%, specificity of 73.3%, positive predictive value of 89.5%, negative predictive value of 84.6%, and diagnostic accuracy rate of 88.2%, and AUC (95% CI) = 0.839 (0.739–0.939).

**Conclusion:**

Combining ECT with CT has a good diagnostic efficacy for metastatic malignant spinal tumors.

## 1. Introduction

Metastasis is a typical manifestation of the progression of malignant tumors to the middle and late stages, and lung, liver, and bones are the most common metastatic sites, with 66.7% of bone metastases belonging to spinal metastasis [1], that is, primary malignant tumors form secondary tumors by invading the spine through vascular, lymphatic, and other routes. Metastatic malignant spinal tumors not only cause patients to experience symptoms such as bone pain but may also trigger functional disorders such as fractures, reducing their quality of survival and increasing the risk of death [2, 3]. Since there is no cure for spinal metastasis, early diagnosis and targeted treatment are important measures to alleviate the clinical symptoms of patients [4], which is of great significance to improve the patient outcome. At this stage, pathological examination is still the gold standard for testing the nature of spinal tumors, but it is traumatic, and biopsy puncture is not feasible in some patients (such as those with coagulation disorder or poor compliance) [5], so minimally invasive and convenient imaging tests are gaining attention in clinic. Computed tomography (CT), X-rays, and emission computed tomography (ECT) are all common imaging modalities for the diagnosis of spinal tumors, and among them, X-ray films can clarify the nature of the lesion based on its morphology, presence or absence of bone destruction, etc., but some lesions that appear abnormal on ECT often require months to be visualized on X-ray films [6], so X-rays are generally used in the initial examination. CT has better sensitivity for the detection of metastatic spine tumors compared to X-rays and is especially valuable for suspected metastatic lesions with X-ray negative and ECT positive, because it is capable of visualizing the involvement of bone trabecula, cortical bone, and surrounding soft tissues. However, the efficacy of CT is also limited by the time that malignant cells invade the spine, and it cannot effectively detect the invaded bone without significant changes in physiological structure and osseous characteristics [7]. Unlike CT and X-rays, ECT evaluates the lesion mainly based on the abnormalities of radionuclides so that the nature of the lesion can be distinguished by the difference in radioactivity concentration, and its early diagnostic efficacy is superior to that of conventional imaging modalities.

According to the published works, the sensitivity of ECT for the diagnosis of metastatic malignant spine tumors ranges from 64.4% to 82.1% [8], and its specificity is relatively low [9], so the atypical lesions should be diagnosed using a combination of multiple imaging tests. Because ECT and CT have differences in imaging mechanism, both of them may give play to the effect of information complementation and improve the clinical detection rate of atypical lesions. By reviewing previous studies, it is found that the literature of combining ECT with CT for the diagnosis of metastatic malignant spine tumors is very few, and although some works explored the diagnostic value of SPECT/CT fusion imaging in single lesion of the spine [10], the sample size was small, causing difficulty in providing a sufficient basis for clinical application. Based on this, 102 patients with malignant tumors were included herein, and the diagnostic value of ECT combined with CT for metastatic malignant spine tumors was analyzed by exploring the detection rate in different malignant tumors, aiming to offer greater theoretical support for clinical practice.

## 2. Materials and Methods

### 2.1. General Data

Inclusion criteria are as follows: (1) the patients had extraskeletal primary malignant tumor; (2) single lesion of spine was found in the patients after whole-body bone imaging; (3) the patients were treated in our hospital in the whole course and had complete clinical data; (4) the patients were at least 18 years old; (5) the patients agreed to undergo puncture biopsy, and the nature of their single lesion in the spine was determined by pathological examination; and (6) the patients received follow-up for over half a year. Exclusion criteria are as follows: (1) the patients had primary malignant tumor of spine; (2) the patients had confirmed metastatic lesions at other sites in addition to the skeletal system; (3) according to the whole-body bone imaging, the patients had other bone abnormal concentrated focus other than single lesion of spine and obvious benign concentrated focus; (4) the patients were under the age of 18; and (5) the nature of patients' single lesion of spine was unclear.

A total of 102 patients with extraskeletal primary malignant tumor treated in our hospital from February 2019 to February 2021 were selected as the subjects. All patients had single lesion of the spine, of which 72 were malignant and 30 were benign according to the results of pathological examination. Among the patients with metastatic malignant tumor of spine, there were 42 females and 30 males, the mean age was (50.29 ± 5.19) years, 18 cases had breast cancer, 23 cases had lung cancer, 17 cases had liver cancer, and 14 cases had prostate cancer; and among the patients with benign tumor of spine, there were 12 females and 18 males, the mean age was (50.13 ± 3.80), 6 cases had breast cancer, 10 cases had lung cancer, 8 cases had liver cancer, and 6 cases had prostate cancer.

### 2.2. Moral Consideration

The study met the principles of World Medical Association Declaration of Helsinki [11], and the study team explained the study purpose, meaning, content, and confidentiality to the patients and asked the patients to sign the informed consent.

### 2.3. Methods

#### 2.3.1. Examination Methods

All patients received the ECT and CT examinations.

ECT: the ECT tester made by GE Medical Systems Israel Ltd. (NMPA registration (I) no. 20163064732) was used, the ^99 m^ TC-MDP tracer agent (provided by Beijing Xinke Sida Pharmaceutical Technology Co. Ltd.; labeling yield: 95%) was administered by intravenous injection, after that, the patients were told to drink more water and urinate more often 3 hours before ECT. During the examination, the patients were in the whole-body imaging position, the low-energy high-resolution collimator was applied, and two probes collected the anterior and posterior views at the same time, with the acquisition matrix of 256 × 512, scanning speed of 20 cm/min, window width of 20%, energy peak of 140 keV, voltage of 120 kV, and the sum of anterior and posterior acquisition counts ≥3.0 M. Then, 7100 A/DI was selected for whole-body bone SPECT scan with anterior and posterior views, and tomographic and local planar views were performed when necessary.

CT: first, X-ray positioning film scanning was performed to determine the scanner field, which centered on the vertebral body of the lesion shown by bone imaging, including 3 adjacent vertebral bodies on either side. After the position was determined, CT scan was performed with the CT scanner (Brilliance CT Big Bore; NMPA (I) 20093300931) made by Philips (China) Investment Co. Ltd., with the matrix of 256 × 256, slice thickness of 5 mm, tube voltage of 130 kV, tube current of 60 mA, automatic exposure tracking, collimator width of 2.5 mm, pitch of 1.5, and rotation time of 0.8 s.

#### 2.3.2. Diagnostic Methods

ECT: (1) criteria for benign lesion: focal uptake was noted in the anterior part of the vertebral bodies, the end plates of the vertebral bodies and the facet joints, and radioactive concentration ≤ anterior superior iliac spine. (2) Criteria for malignant lesion: focal uptake was noted in the posterior part of the vertebral bodies, pedicle of vertebral arch, and radioactive concentration > anterior superior iliac spine.

CT: (1) criteria for benign lesion: hyperostosis, osteophyte formation at the edge of the vertebral bodies; coarse and indistinct cartilaginous surfaces of the vertebral bodies; degeneration of the intervertebral disc, narrowing of intervertebral space; facet wear with blurring of articular surfaces; spondylolisthesis, scoliosis, and spondylolysis. (2) Criteria for malignant lesion: bone resorptive lesions without sclerotic margins; osteoblastic focus; bone resorptive-osteoblastic mixed focus.

### 2.4. Efficacy Analysis

By the blind method, 2 experienced nuclear medicine physicians interpreted the ECT and CT images of 102 patients, and the 102 lesions were classified as benign (both ECT and CT showed benign) and malignant (ECT or CT showed malignant). The diagnostic efficacy of ECT and CT was calculated by comparing the physicians' interpretation results and pathological findings and plotting the ROC cures as follows. (1) Sensitivity: number of true positive cases/(number of true positive cases + number of false negative cases) × 100%; (2) specificity: number of true negative cases/(number of true negative cases + number of false positive cases) × 100%; (3) positive predictive value (PPV): number of true positive cases/(number of true positive cases + number of false positive cases); (4) negative predictive value (NPV): number of true negative cases/(number of false negative cases + number of true negative cases).

### 2.5. Statistical Processing

In this study, the data processing software was SPSS20.0, the picture drawing software was GraphPad Prism 7 (GraphPad Software, San Diego, USA), the items included were enumeration data and measurement data, the methods used were the *X*^2^ test and *t*-test, and differences were considered statistically significant at *P* < 0.05.

## 3. Results

### 3.1. Comparison of ECT and CT Diagnostic Results

Tables 1–3 showed the diagnostic results of ECT, CT, and their combination.

### 3.2. Comparison of Detection Rates of Metastatic Malignant Spinal Tumors in Different Malignancies

ECT + CT had a detection rate of 100% of metastatic malignant spinal tumors in breast cancer and lung cancer, 94.1% in liver cancer, and 78.6% in prostate cancer, respectively (see Table 4).

### 3.3. Analysis of Diagnostic Efficacy of ECT and CT

**Figure 1:**
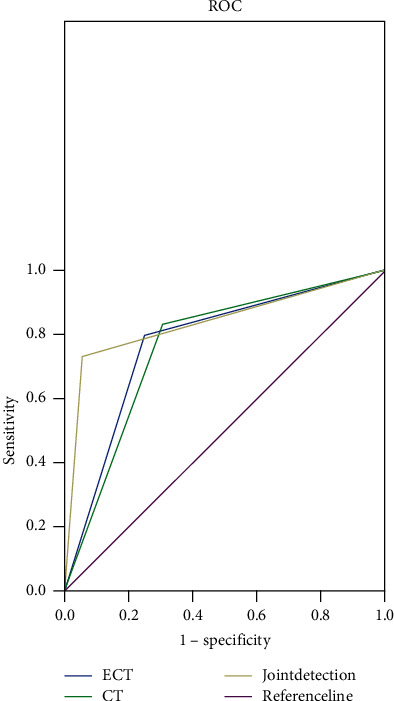
ROC curves of ECT, CT, and their combination.

ECT combined with CT had a diagnostic sensitivity of 94.4%, specificity of 73.3%, PPV of 89.5%, NPV of 84.6%, and diagnostic accuracy rate of 88.2% (see Table 5). Also, according to the ROC curves, the combined diagnosis obtained AUC (95% CI) = 0.839 (0.739–0.939) (see Figure 1).

## 4. Discussion

When the malignant tumors progress to the middle and late stages, tumor cells will metastasize to other tissues through lymphatic, vascular, body cavity, and other routes, and although different malignant tumors may metastasize to different tissues, the metastatic lesions more likely occur in tissues with rich blood supply [12, 13], so bones are one of the most common tissues with distant metastasis. It has been reported in relevant investigations that breast cancer and prostate cancer patients are most likely to present with bone metastasis, 80.0% of female breast cancer patients died due to bone metastasis [14] and 90.0% of male prostate cancer patients had increased risk of death [15], and therefore, it is extremely important to enhance the prevention and treatment of bone metastasis. Because the red marrow is mainly distributed in the axial bone, which has a capillary network suitable for tumor embolus growth and no venous valve inside its venous network, so any factors that trigger the elevation of pelvic and thoracic pressure will cause the tumor embolus to enter the venous plexus [16]. Hence, the spine is the most susceptible skeletal tissue to metastasis, and early screening for spinal metastasis is the focus in the prevention and treatment of bone metastasis. At this stage, pathological examination is still the gold standard to examine the nature of spinal tumors, but not all patients can undergo puncture or surgical sampling, and it is difficult to obtain the pathological results of all lesions in practice, so some scholars advocate the combination of multiple imaging examinations to screen metastatic malignant spinal tumors and the implementation of regular follow-up of patients to avoid non-necessary pathological examinations [17, 18].

X-rays, CT, and ECT are the most common examination modalities for metastatic spine tumors, in which X-rays and CT reflect the presence of metastasis based on the osteolytic changes of bone tissue and decalcification condition at the lesion site [19], but bone density changes on X-rays can be found only in case of more than 30.0% of the decalcification condition [20], so the possibility of tumor metastasis cannot be excluded for those without abnormalities in X-rays and further CT examination is needed. This study showed that CT had a sensitivity of 69.4% and NPV of 53.2% for the diagnosis of metastatic malignant spine tumors, which was due to the fact that although CT can clearly demonstrate the anatomy of the spine, it fails to sufficiently show the overlapping sites of lesions and microlesions as there are more overlapping sites in the spine, and diseases such as osteoporosis may also affect the accuracy of the results [21], resulting in a low NPV. After applying ECT examination, a total of 68 metastatic malignant spine tumors were detected, and the diagnostic sensitivity, accuracy, and NPV obviously rose, indicating that ECT combined with CT can play a role of mechanism complementation and improve the diagnostic accuracy for spine tumors. ECT is based on the technique of radioactive nuclear element tracing, which can make the diseased tissue present different radioactivity concentrations from normal tissue by the way of injecting radiopharmaceuticals, thus helping physicians to judge the metastasis of malignant tumors [22, 23]. Because of the wide distribution of glands within the breast, the detection rate of combined examination of metastatic malignant spinal tumors in breast cancer is 100.0%. Although the detection rate in lung cancer is also 100.0%, the mechanism is not clear and may be related to the rich blood vessels. In comparison, the detection rates in liver cancer and prostate cancer were relatively low, which were respectively 94.1% and 78.6%. Liu et al. adopted ECT examination alone to obtain a bone metastasis detection rate of 80.0% in liver cancer and 50.0% in prostate cancer [24], demonstrating that the combined examination has application value in spinal metastasis of different types of malignancies.

To sum up, ECT, as the most common nuclear medicine examination method, achieves a sensitivity that has been recognized by the academic community [25], but there is still an important meaning in combining ECT with CT for comprehensive judgment in most malignant tumors. ECT examination can be applied to localized subtle lesions, and lesions that are difficult to define by ECT can be morphologically distinguished with CT to confirm whether or not they are metastatic malignant spinal tumors. If the patient has significant bone destruction, the CT examination may be used to make up for ECT. The study results showed that combining ECT with CT obtained AUC (95% CI) = 0.839 (0.739–0.939), implying a good diagnostic efficacy of the combined diagnosis for metastatic malignant spine tumors. Besides, this diagnostic modality is convenient and high-efficient, which can greatly improve the efficacy of diagnosing metastatic malignant spine tumors.

## Figures and Tables

**Table 1:** Diagnostic results of ECT.

ECT	Pathological examination		Total
	Malignant	Benign	
Malignant	54	6	60
Benign	18	24	42
Total	72	30	102

**Table 2:** Diagnostic results of CT.

CT	Pathological examination		Total
	Malignant	Benign	
Malignant	50	5	55
Benign	22	25	47
Total	72	30	102

**Table 3:** Diagnostic results of ECT combined with CT.

ECT + CT	Pathological examination		Total
	Malignant	Benign	
Malignant	68	8	76
Benign	4	22	26
Total	72	30	102

**Table 4:** Comparison of detection rates of metastatic malignant spinal tumors in different malignancies.

	Breast cancer	Lung cancer	Liver cancer	Prostate cancer	Total
Number of cases	18	23	17	14	72
Number of positive cases	18	23	16	11	68
Detection rate	100.0%	100.0%	94.1%	78.6%	94.4%

**Table 5:** Analysis of diagnostic efficacy of ECT and CT.

Group	Sensitivity (%)	Specificity (%)	PPV (%)	NPV (%)	Accuracy rate (%)
ECT	75.0 (54/72)	80.0 (24/30)	90.0 (54/60)	57.1 (24/42)	76.5 (78/102)
CT	69.4 (50/72)	83.3 (25/30)	90.9 (50/55)	53.2 (25/47)	73.5 (75/102)
ECT + CT	94.4 (68/72)	73.3 (22/30)	89.5 (68/76)	84.6 (22/26)	88.2 (90/102)

## Data Availability

The data used to support the findings of this study are available on reasonable request from the corresponding author.

## References

[1] Ge L., Arul K., Mesfin A. (2019). Spinal cord injury from spinal tumors: prevalence, management, and outcomes. *World Neurosurgery*.

[2] Maj E., Szemplińska B., Prokopienko W. (2020). Role of diffusion tensor imaging parameters in the characterization and differentiation of infiltrating and non-infiltrating spinal cord tumors: preliminary study. *Clinical Neuroradiology*.

[3] Muraoka S., Yamane K., Haruo Misawa H. (2021). Assessment of the concordance rate between intraoperative pathological diagnosis and the final pathological diagnosis of spinal cord tumors. *Acta Medica Okayama*.

[4] Ottenhausen M., Ntoulias G., Bodhinayake I. (2019). Intradural spinal tumors in adults-update on management and outcome. *Neurosurgical Review*.

[5] Yuan Y., Lang N., Yuan H. S. (2020). [CT spectral curve in differentiating spinal tumor metastasis and infections]. *Beijing Da Xue Xue Bao Yi Xue Ban*.

[6] Wang Y., Yin A., Bian T. (2021). Observation of efficacy of internet-based chronic disease management model combined with modified therapy of bushenyiliu decoction in treating patients with type 2 diabetes mellitus and prostate cancer and its effect on disease control rate. *Evidence-based Complementary and Alternative Medicine*.

[7] Safaee M. M., Burke J. F., Dalle Ore C. L. (2021). Evaluating the clinical utility and cost of imaging strategies in adults with newly diagnosed primary intradural spinal tumors. *World Neurosurgery*.

[8] Abd-El-Barr M. M., Huang K. T., Moses Z. B., Iorgulescu J. B., Chi J. H. (2018). Recent advances in intradural spinal tumors. *Neuro-Oncology*.

[9] Subramanian A., Nair B. R., Rajshekhar V. (2021). Functional outcomes and temporal profile of recovery in patients with intradural extramedullary spinal cord tumors with poor nurick grade. *World Neurosurgery*.

[10] Bilsky M. H., Gokaslan Z., Shin J. H., Dea N., Ynoe de Moraes F. (2021). Introduction. Treatment of spinal cord and spinal axial tumors. *Neurosurgical Focus*.

[11] World Medical Association (2013). World medical association declaration of Helsinki. *JAMA*.

[12] Zhuo H., Zhou Y., Chai X., Chang Q., Rao G. (2019). The application of ultrasonic bone curette in laminoplasty of spinal canal after resection of intraspinal tumors. *Zhongguo Xiu Fu Chong Jian Wai Ke Za Zhi*.

[13] Formo M., Halvorsen C. M., Dahlberg D. (2018). Minimally invasive microsurgical resection of primary, intradural spinal tumors is feasible and safe: a consecutive series of 83 patients. *Neurosurgery*.

[14] Ma Y., Chen L., Li X. (2021). Rationally integrating peptide-induced targeting and multimodal therapies in a dual-shell theranostic platform for orthotopic metastatic spinal tumors. *Biomaterials*.

[15] Jakubovic R., Ruschin M., Tseng C. L., Pejović-Milić A., Sahgal A., Yang V. X. D. (2019). Surgical resection with radiation treatment planning of spinal tumors. *Neurosurgery*.

[16] Kobayashi Y., Kawabata S., Nishiyama Y. (2019). Changes in sagittal alignment after surgical excision of thoracic spinal cord tumors in adults. *Spinal Cord*.

[17] Pessina F., Navarria P., Carta G. A. (2018). Long-term follow-up of patients with metastatic epidural spinal cord compression from solid tumors submitted for surgery followed by radiation therapy. *World Neurosurgery*.

[18] Sun J. J., Yang J., Xie J. C. (2019). Comparative clinical study on seldom segment with multiple segment intramedullary primary spinal cord tumors. *Beijing da xue xue bao. Yi xue ban = Journal of Peking University. Health sciences*.

[19] Tsunoda K. (2021). Spinal cord tumors:classification, treatment, and prognosis. *Noshinkeigeka*.

[20] Xiong W., Xu Y., Fang Z., Li F. (2018). Total en bloc spondylectomy for lumbar spinal tumors by paraspinal approach. *World Neurosurgery*.

[21] Klimov V. S., Kel’makov V. V., Chishchina N. V., Evsyukov A. V. (2018). Effectiveness of intraoperative monitoring of motor evoked potentials for predicting changes in the neurological status of patients with cervical spinal cord tumors in the early postoperative period. *Voprosy neirokhirurgii imeni N.N. Burdenko*.

[22] Benesch M., Nemes K., Neumayer P. (2020). Spinal cord atypical teratoid/rhabdoid tumors in children: clinical, genetic, and outcome characteristics in a representative European cohort. *Pediatric Blood and Cancer*.

[23] Hongyu W., Dong C., Wu J., Zhu Y., Ma H. (2020). Total en bloc spondylectomy combined with the satellite rod technique for spinal tumors. *Journal of Orthopaedic Surgery and Research*.

[24] Liu P., Liang Y., Bian C. (2020). Diagnostic accuracy of MR, CT, and ECT in the differentiation of neoplastic from nonneoplastic spine lesions. *Asia-Pacific Journal of Clinical Oncology*.

[25] Zhang H.-R., Qiao R.-Q., Yang X.-G., Hu Y.-C. (2020). A multicenter, descriptive epidemiologic survey of the clinical features of spinal metastatic disease in China. *Neurological Research*.

